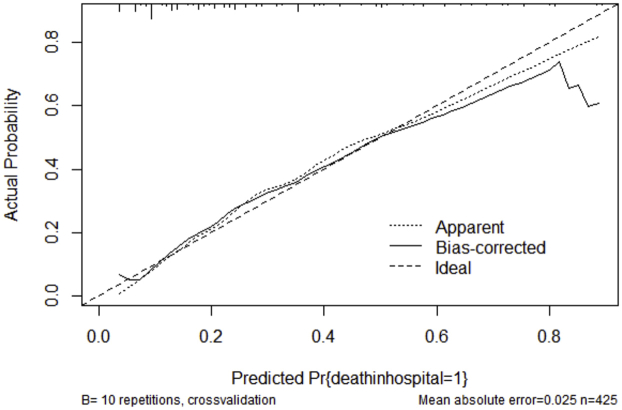# North American COVID-19 Myocardial Infarction (NACMI) Risk Score for Prediction of In-Hospital Mortality

**DOI:** 10.1016/j.jscai.2022.100404

**Published:** 2022-07-09

**Authors:** Payam Dehghani, Christian W. Schmidt, Santiago Garcia, Brynn Okeson, Cindy L. Grines, Avneet Singh, Rajan A.G. Patel, Jose Wiley, Wah Wah Htun, Keshav R. Nayak, M. Chadi Alraies, Nima Ghasemzadeh, Laura J. Davidson, Deepak Acharya, Jay Stone, Tareq Alyousef, Brian C. Case, Xuming Dai, Abdul Moiz Hafiz, Mina Madan, Faoruc A. Jaffer, Jay S. Shavadia, Ross Garberich, Akshay Bagai, Jyotpal Singh, Herbert D. Aronow, Nestor Mercado, Timothy D. Henry

**Affiliations:** a Prairie Vascular Research Inc, Regina, Saskatchewan, Canada; b Minneapolis Heart Institute Foundation, Minneapolis, Minnesota; c Northside Cardiovascular Institute, Atlanta, Georgia; d Long Island Jewish Medical Center and North Shore University Hospital, New Hyde Park, New York; e Ochsner Medical Center, New Orleans, Louisiana; f Albert Einstein College of Medicine, Montefiore Health System, New York, New York; g Gundersen Health System, La Crosse, Wisconsin; h Department of Cardiology, Scripps Mercy Hospital, San Diego, California; i DMC Harper University Hospital, Detroit, Michigan; j Georgia Heart Institute, Gainesville, Georgia; k Northwestern University, Feinberg School of Medicine, Chicago, Illinois; l University of Arizona Sarver Heart Center, Tucson, Arizona; m Community Medical Center (RWJ Barnabas Health), Toms River, New Jersey; n Cook County Health and Hospitals System, Chicago, Illinois; o MedStar Washington Hospital Center, Washington, District of Columbia; p NewYork-Presbyterian Queens, Flushing, New York; q Southern Illinois University School of Medicine, Springfield, Illinois; r Schulich Heart Centre, Sunnybrook Health Sciences Centre, Toronto, Ontario, Canada; s Massachusetts General Hospital, Boston, Massachusetts; t Royal University Hospital (Saskatchewan Health), University of Saskatchewan, Saskatoon, Saskatchewan, Canada; u St. Michael’s Hospital, Toronto, Ontario, Canada; v Alpert Medical School of Brown University and Lifespan Cardiovascular Institute, Providence, Rhode Island; w University of New Mexico, Albuquerque, New Mexico; x The Carl and Edyth Lindner Center for Research and Education, The Christ Hospital, Cincinnati, Ohio

**Keywords:** COVID-19, myocardial infarction, risk score, mortality

## Abstract

**Background:**

In-hospital mortality in patients with ST-segment elevation myocardial infarction (STEMI) is higher in those with COVID-19 than in those without COVID-19. The factors that predispose to this mortality rate and their relative contribution are poorly understood. This study developed a risk score inclusive of clinical variables to predict in-hospital mortality in patients with COVID-19 and STEMI.

**Methods:**

Baseline demographic, clinical, and procedural data from patients in the North American COVID-19 Myocardial Infarction registry were extracted. Univariable logistic regression was performed using candidate predictor variables, and multivariable logistic regression was performed using backward stepwise selection to identify independent predictors of in-hospital mortality. Independent predictors were assigned a weighted integer, with the sum of the integers yielding the total risk score for each patient.

**Results:**

In-hospital mortality occurred in 118 of 425 (28%) patients. Eight variables present at the time of STEMI diagnosis (respiratory rate of >35 breaths/min, cardiogenic shock, oxygen saturation of <93%, age of >55 ​years, infiltrates on chest x-ray, kidney disease, diabetes, and dyspnea) were assigned a weighted integer. In-hospital mortality increased exponentially with increasing integer risk score (Cochran-Armitage χ^2^, *P* ​< ​.001), and the model demonstrated good discriminative power (c-statistic ​= ​0.81) and calibration (Hosmer-Lemeshow, *P* ​= ​.40). The increasing risk score was strongly associated with in-hospital mortality (3.6%-60% mortality for low-risk and very high–risk score categories, respectively).

**Conclusions:**

The risk of in-hospital mortality in patients with COVID-19 and STEMI can be accurately predicted and discriminated using readily available clinical information.

## Highlights

• From 425 patients with COVID-19 and STEMI, in-hospital mortality was 28%
• Eight variables predict mortality risk for patients with COVID-19 and STEMI
• Cumulative risk scores have strong discriminative ability for predicting mortality
• Mortality risk should be assessed in incoming patients with COVID-19 and STEMI using readily available clinical variables

## Central Illustration

**Figure:**
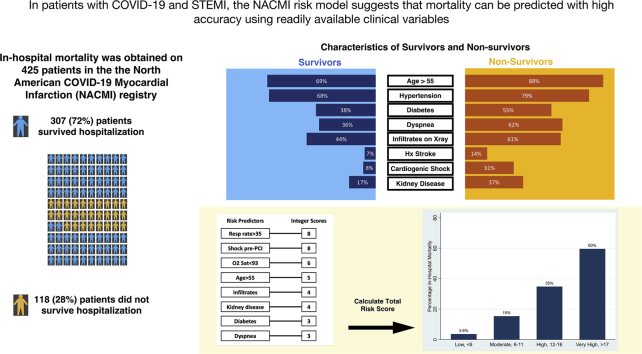

## Introduction

In-hospital mortality in patients with ST-segment elevation myocardial infarction (STEMI) is 4 to 8 times higher in those with COVID-19 than in those without COVID-19.^1^, ^2^, ^3^, ^4^ In patients with COVID-19, STEMI may result from various mechanisms, including plaque rupture, prothrombotic and proinflammatory states, microthrombi, and myocarditis. Delayed presentation and extracardiac morbidity (eg, stroke, pulmonary embolism, pneumonia, excess thrombus burden, multiorgan failure, and higher rates of shock) also contribute to this mortality excess.

Multiple risk scores have been developed and validated to predict in-hospital mortality in patients with COVID-19 but none exist for those who are also hospitalized with STEMI. The pandemic has uncovered critical stress points within the health care system, forcing clinicians to triage patients who are more likely to survive so that resources can be allocated accordingly. Furthermore, the variables associated with the increased death rate in this population are not known. Therefore, we used data from the North American COVID-19 Myocardial Infarction (NACMI) registry to develop a universally applicable, easy-to-employ risk score to predict in-hospital mortality in patients with COVID-19 hospitalized with STEMI.

## Materials and methods

### Data source

NACMI is a prospective, investigator-initiated, observational registry enrolling patients with STEMI and suspected or confirmed COVID-19 at 64 clinical sites in the United States and Canada. The ethical approval process, registry design, and description of patient characteristics and outcomes have been published previously.^1^^,^^5^ Briefly, patients with STEMI and confirmed COVID-19 and those with STEMI and suspected COVID-19 who were subsequently deemed negative were enrolled from April 2020 to June 2021. The registry captured demographic information, descriptors at presentation with STEMI, and clinical outcomes.^1^^,^^5^ Only patients with confirmed COVID-19 and STEMI were included in this analysis.

### Participants

Consecutive adults aged ≥18 ​years with the following were included: (1) ST-segment elevation in at least 2 contiguous leads (or new onset left bundle branch block); (2) a clinical correlate of myocardial ischemia (eg, chest pain, dyspnea, cardiac arrest, shock, mechanical ventilation); and (3) confirmed SARS-CoV-2 infection, identified with any commercially available test administered during or within 4 ​weeks before the index hospitalization for STEMI. Patients without available vital status were excluded. Patients with a “do not resuscitate status” or multiple futility markers on admission, in whom percutaneous coronary intervention (PCI) was appropriate, were also excluded. The protocol was approved by each local institutional review board. Informed consent was waived.

### Outcome

The primary outcome measure was in-hospital all-cause mortality.

### Predictors

For the purposes of developing this model, the moment of prognostication (T ​= ​0) was defined as the time of the index electrocardiogram demonstrating ST-segment elevation, with the end of follow-up defined as hospital discharge or death. As the “Transparent Reporting of a multivariable prediction model for Individual Prognosis or Diagnosis” statement recommends, we only retrospectively analyzed variables at T ​= ​0 and, therefore, excluded intermediary variables that occurred thereafter, such as percutaneous coronary interventions or mechanical ventilation.^6^ Variables at T ​= ​0 included patient demographic characteristics, risk factors and comorbidities, and clinical presentation (Supplemental Table S1).

### Sample size and missing data

**Figure. 1:**
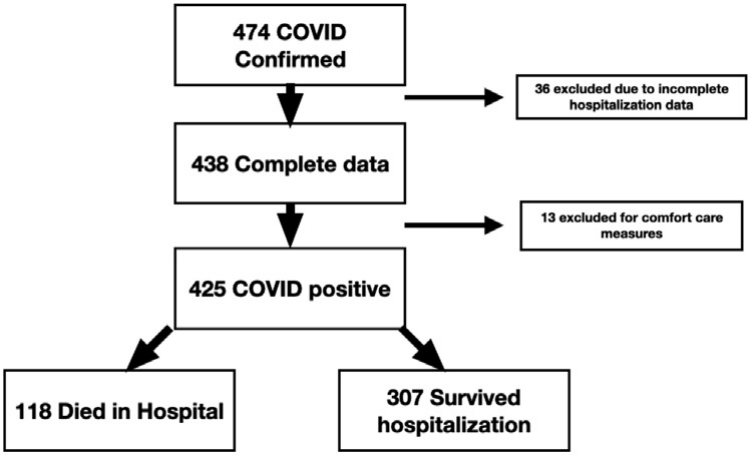
Flow chart of NACMI patients included in this analysis. Of 474 patients, 36 had incomplete data available and 13 were excluded for comfort care measures, resulting in 425 patients with COVID-19 and STEMI included in this study. Of these, 118 patients died in the hospital, representing 28% mortality, whereas 307 patients survived hospitalization. NACMI, North American COVID-19 Myocardial Infarction; STEMI, ST-segment elevation myocardial infarction.

Only patients with complete data were included in the analyses. Furthermore, any patients with inadequate detail of their entered data were excluded for comfort care measures (Figure 1).

**Central Illustration:**
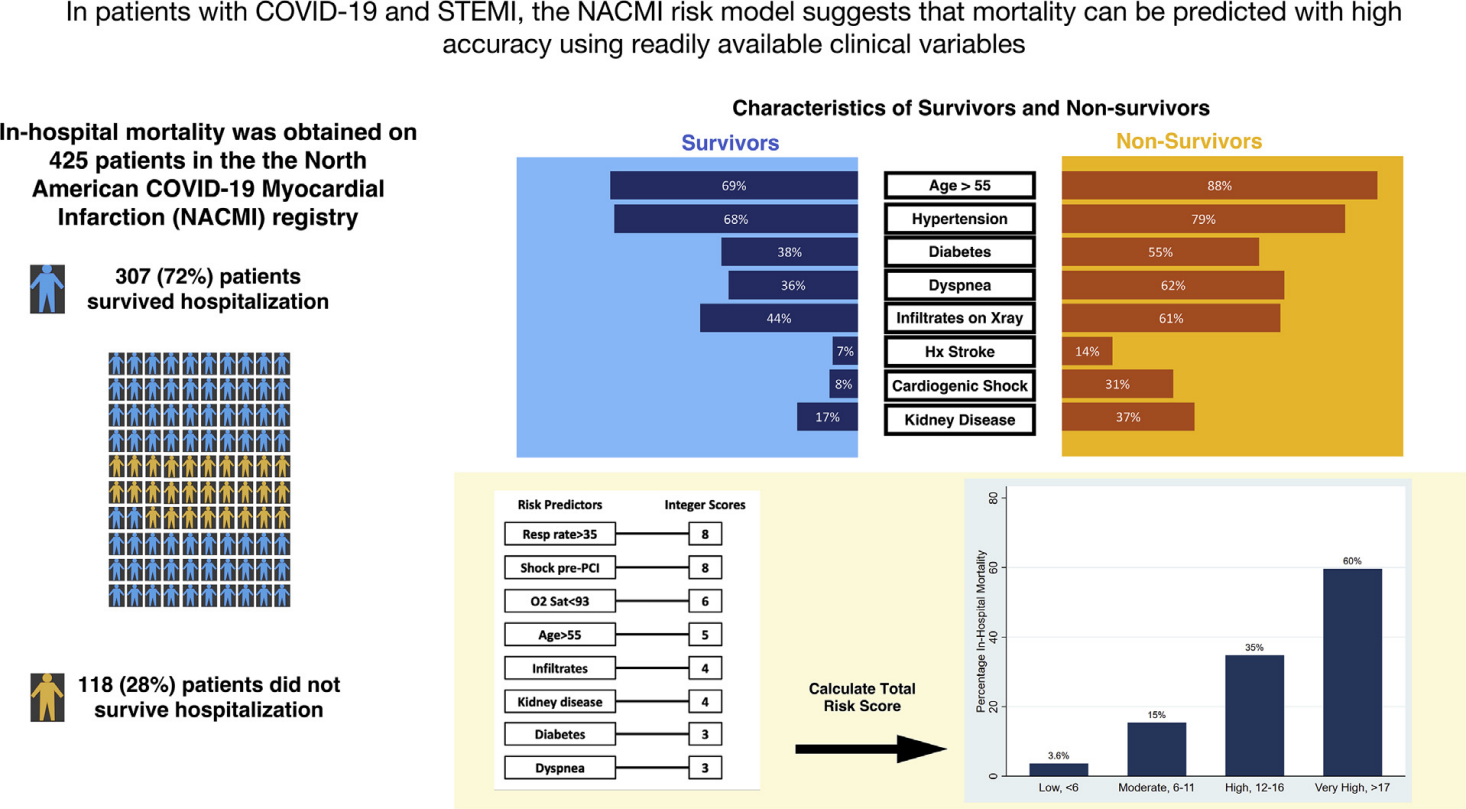
Demographics and clinical characteristics of patients with COVID-19 and STEMI contributing to the NACMI (North American COVID-19 Myocardial Infarction) Risk Score Calculation for Prediction of In-Hospital Mortality.

### Statistical analysis and characterization of risk groups

In developing this multivariable prediction model for prognosticating in-hospital mortality in patients with COVID-19 and STEMI, we used the guidelines put forward by the Transparent Reporting of a multivariable prediction model for Individual Prognosis or Diagnosis statement.^6^ The data coordinating center at the Minneapolis Heart Institute Foundation had full access to the data set and performed the statistical analysis. Descriptive statistics are presented as counts and percentages for discrete variables, mean ​± ​standard deviation for normally distributed continuous variables, and median (interquartile range) for nonnormally distributed continuous variables. Simple imputation was performed to replace the missing data. Candidate variables were selected on the basis of prior knowledge and included 24 demographic, clinical, and treatment variables (Supplemental Table S1). Univariable logistic regression was performed using candidate variables to predict in-hospital mortality. Multivariable logistic regression was then performed using backward stepwise selection on predictors significant in univariable models at a level of *P* ​< ​.1 to identify independent predictors of in-hospital mortality. The 8 variables included in the final multivariable logistic regression model were assigned a weighted integer coefficient value, such that an integer of 1 was assigned for every 0.5 odds ratio value. The final risk score represents the sum of the integer coefficients. The final score was separated into the following sum totals corresponding to the quartiles of the risk score distribution: <6 (low-risk group), 6 to 11 (moderate-risk group), 12 to 16 (high-risk group), and >17 (very high–risk group). Model calibration was assessed by plotting expected vs observed probabilities and the Hosmer-Lemeshow goodness-of-fit statistic.^7^ Model discrimination was assessed with the apparent c-statistic and optimism-corrected c-statistic estimated from 10-fold cross validation. All analyses were performed using Stata version 15.1 and R version 4.1.3. Cross validation was performed using the rms package in R (v. 6.2-0).^8^

## Results

### Participants

Between April 2020 and June 2021, 474 participants with COVID-19 and STEMI were enrolled in the registry. Of these, 36 had incomplete data and 13 were excluded because of comfort care measures in place, resulting in 425 participants (Figure 1). Of these, 118 (28%) died in the hospital (Figure 1). Patient characteristics in the overall population and according to in-hospital vital status are presented in Table 1. Most patients were men (72%), aged 56 to 75 ​years, and more likely to be of minority ethnicity (21% Hispanic, 18% Black, 7% Asian), with Caucasians representing only 46% of the patients. Compared with those who did not survive hospitalization, survivors were less likely to have diabetes (38% vs 55%), hypertension (68% vs 79%), and a history of stroke (7% vs 14%). Chest pain was the most common presenting symptom (57%), but dyspnea was also common (49%), and 43% of patients had infiltrates on chest x-ray. A sizable proportion presented with high-risk conditions pre-PCI, including cardiogenic shock (16%) and cardiac arrest (9%). Of 152 patients in the no-PCI group, 44 did not undergo angiography (mortality 43%), 61 patients underwent angiography but did not have an identifiable culprit lesion (mortality 48%), and 26 patients underwent angiography with a culprit lesion identified but did not undergo PCI (mortality 23%). The status of the culprit lesion was unclear in the remaining 21 patients, with corresponding mortality of 38%. A risk score with the inclusion of the management variables, including PCI and mechanical ventilation, is included in the supplementary files (Supplemental Tables S1 and S2).

**Table 1:** Characteristics of patients with COVID-19 and STEMI.

Variable	Total (N ​= ​425)	Survivors (n ​= ​307)	Nonsurvivors (n ​= ​118)	*P* value
Female sex	119 (28)	84 (27)	35 (30)	.6
Age group, y				<.001
18-55	110 (26)	96 (31)	14 (12)	
56-65	132 (31)	96 (31)	36 (31)	
66-75	109 (26)	68 (22)	41 (35)	
76-85	60 (14)	38 (12)	22 (19)	
>85	14 (3)	9 (3)	5 (4)	
Race/ethnicity				.4
White	192 (46)	145 (48)	47 (41)	
Black	76 (18)	56 (19)	20 (17)	
Asian	27 (7)	16 (5)	11 (9)	
Hispanic	87 (21)	60 (20)	27 (23)	
Indigenous	9 (2)	5 (2)	4 (3)	
Other	24 (6)	17 (6)	7 (6)	
Weight, kg	83.4 (71-100.2)	85 (72-101)	80 (68-99)	.058
Body mass index, kg/m^2^	28.8 (25-32.6)	29 (25-33)	28 (24-32)	.14
History of CAD	97 (25)	69 (22)	28 (24)	.8
Previous PCI	52 (14)	41 (13)	11 (9)	.3
Previous MI	51 (13)	39 (13)	12 (10)	.5
Previous CABG	19 (5)	11 (4)	8 (7)	.2
Hypertension	292 (71)	204 (68)	88 (79)	.032
Dyslipidemia	178 (45)	127 (41)	51 (43)	.7
Diabetes	182 (46)	117 (38)	65 (55)	.002
Previous stroke/TIA	33 (9)	19 (7)	14 (14)	.033
Smoking history	183 (46)	140 (46)	43 (36)	.088
Current smoker	80 (20)	64 (22)	16 (15)	.085
History of CHF	60 (16)	44 (16)	16 (16)	>.9
Mechanical ventilation	113 (28)	45 (15)	68 (58)	<.001
Any MCS	56 (13)	22 (7)	34 (29)	<.001
COVID-19 symptoms				
Dyspnea	207 (49)	134 (44)	73 (62)	<.001
Chest pain	241 (57)	203 (66)	38 (32)	<.001
Syncope	14 (3)	8 (3)	6 (5)	.2
Abnormal chest x-ray findings				
Infiltrates	181 (43)	109 (36)	72 (61)	<.001
Pleural effusion	38 (9)	24 (8)	14 (12)	.2
Cardiomegaly	35 (8)	23 (7)	12 (10)	.4
Cardiac arrest pre-PCI	35 (9)	20 (7)	15 (15)	.013
Cardiogenic shock pre-PCI	61 (16)	25 (8)	36 (31)	<.001
In-hospital presentation	29 (7)	15 (5)	14 (12)	.011
D2B, min	74 (50-120)	73 (52-113)	81 (44-130)	.6
Ejection fraction, %	45 (34-55)	45 (35-55)	36 (29-55)	.011
O_2_ saturation, %	96 (94-99)	97 (95-99)	94 (89-96)	<.001
Respiratory rate, breaths/min	20 (18-24)	20 (18-22)	23 (20-28)	<.001
Baseline creatinine level, mg/dL^a^	1.05 (0.85-1.45)	1.07 (0.85-1.69)	1.37 (0.98-2.23)	.004
Kidney disease^a^	97 (23)	53 (17)	44 (37)	<.001
Kidney disease category				<.001
Baseline creatinine level <1.5 ​mg/dL	314 (75)	244 (81)	70 (61)	
Baseline creatinine level 1.5-2.0 ​mg/dL	45 (11)	26 (9)	19 (17)	
Baseline creatinine level >2.0 ​mg/dL	58 (14)	33 (11)	25 (22)	
Reperfusion strategy				<.001
Thrombolytics	15 (4)	10 (3)	5 (4)	
Primary PCI	259 (62)	206 (68)	53 (46)	
Facilitated/rescue PCI	11 (3)	9 (3)	2 (2)	
Medical therapy	79 (19)	42 (14)	37 (32)	
CABG	7 (2)	7 (2)	0 (0)	
No angiogram	50 (12)	31 (10)	19 (16)	
Culprit artery^b^				
LMCA	2 (0.6)	2 (0.7)	0 (0)	
LAD/diagonal	112 (31)	90 (33)	22 (24)	
LCx/OM/PDA	21 (6)	16 (6)	5 (6)	
RCA/PDA	90 (25)	72 (27)	18 (20)	
Bypass graft	0	0	0	
Ramus	1 (0.3)	1 (0.4)	0 (0)	
Multiple	61 (17)	49 (18)	12 (13)	
No culprit	75 (21)	41 (15)	34 (37)	
No. of stents				
1	126 (54)	104 (55)	22 (51)	
2	73 (31)	60 (32)	13 (30)	
≥3	33 (14)	25 (13)	8 (19)	
Drug-eluting stent	231 (98)	190 (98)	41 (98)	

Values are n (%) or median (IQR). CABG, coronary artery bypass grafting; CAD, coronary artery disease; CHF, congestive heart failure; D2B, door-to-balloon; LAD, left anterior descending; LCx, left circumflex; LMCA, left main coronary artery; MCS, mechanical circulatory support; MI, myocardial infarction; OM, obtuse marginal; PCI, percutaneous coronary intervention; PDA, posterior descending artery; RCA, right coronary artery; STEMI, ST-segment elevation myocardial infarction; TIA, transient ischemic attack.

a Kidney disease was defined as creatinine level of >1.5 ​mg/dL on presentation.

b Of patients with angiography and identifiable culprit.

### Model development, specification, and performance

**Figure. 2:**
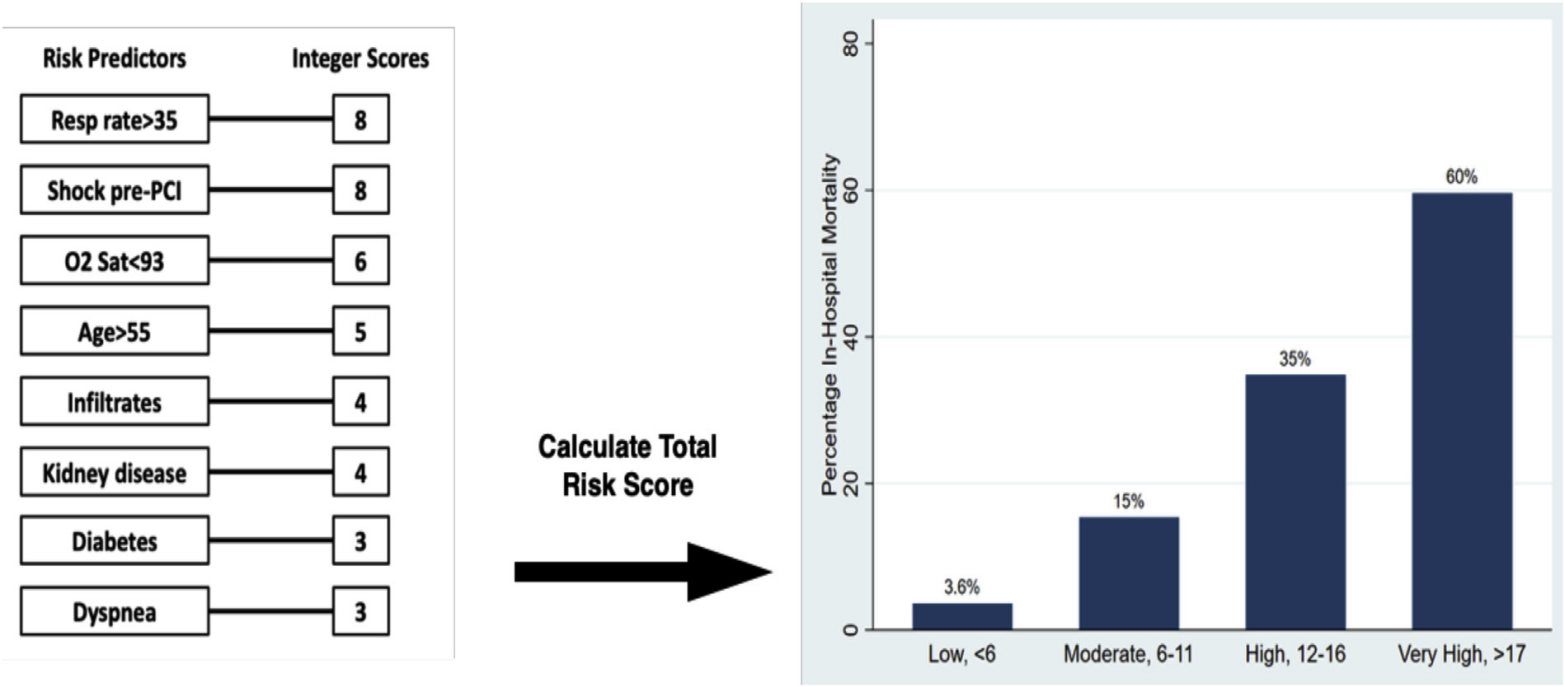
NACMI risk score calculation for prediction of in-hospital mortality. Of the 8 predictors of in-hospital mortality, the odds ratios are given a weighted “integer score.” The cumulation of these integer scores provides a risk value of in-hospital mortality, with <6 representing low risk, 6 to 11 representing moderate risk, 12 to 16 representing high risk, and >17 representing very high risk. NACMI, North American COVID-19 Myocardial Infarction; PCI, percutaneous coronary intervention; Resp, Respiratory; Sat, Saturation.

**Supplemental Figure S1:**
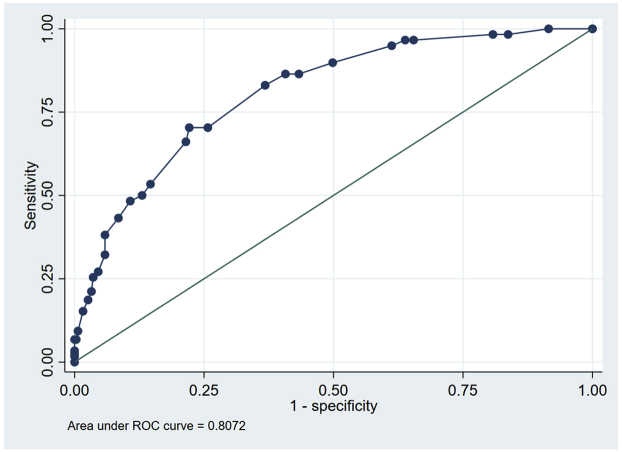


**Supplemental Figure S2:**
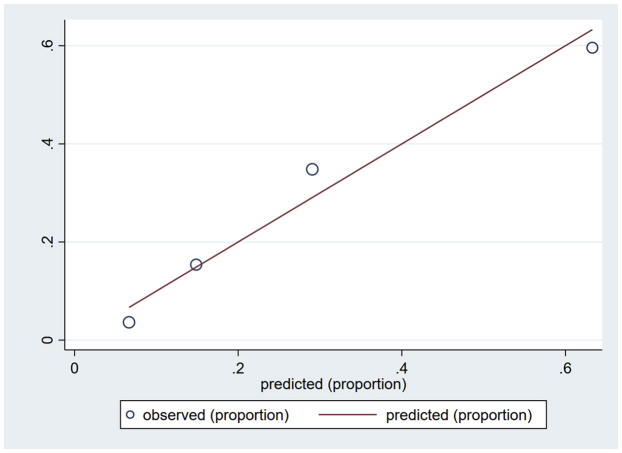


Variables associated with in-hospital mortality in univariable comparisons are shown in Supplemental Table S1. Of the 24 variables explored, 8 associated with in-hospital mortality were incorporated into the multivariable logistic regression (Table 2). When pooled into a risk score, an increasing score value was associated with higher odds of in-hospital mortality (Cochran-Armitage χ^2^, *P* ​< ​.001). The model demonstrated good discriminative ability (apparent c-statistic ​= ​0.81, optimism-corrected c-statistic ​= ​0.78, Supplemental Figure S1) and calibration (Supplemental Figures S2 and S3). The risk score group distribution (based on observed in-hospital mortality rate) was as follows: 110 (26%) corresponded to the low-risk group (3.6% risk of mortality), 104 (24%) corresponded to the moderate-risk group (15% mortality), 112 (26%) corresponded to the high-risk group (35% mortality), and 99 (23%) corresponded to the very high–risk group (60% mortality) (Figure 2). The Hosmer-Lemeshow statistic was χ^2^ of 4.02 (*P* ​= ​.40), and the observed vs predicted probabilities are presented in Supplemental Figures S2 and S3.

**Table 2:** Independent predictors of in-hospital mortality in patients with COVID-19 and STEMI.

Multivariable analysis	Integer score	Coefficient	Odds ratio	95% CI	*P* value
Respiratory rate >35 breaths/min	8	1.42	4.12	1.29-13.13	.017
Shock pre-PCI	8	1.43	4.17	2.18-7.97	<.001
O_2_ saturation <93%	6	1.09	2.97	1.66-5.33	<.001
Age >55 ​y	5	0.90	2.45	1.25-4.80	.009
Infiltrates on chest x-ray	4	0.78	2.17	1.31-3.61	.003
Kidney disease (creatinine level >1.5 ​mg/dL)	4	0.63	1.87	1.07-3.27	.028
Diabetes	3	0.51	1.67	1.01-2.76	.046
Dyspnea	3	0.44	1.56	0.94-2.59	.088

PCI, percutaneous coronary intervention; STEMI, ST-segment elevation myocardial infarction.

## Discussion

Using the NACMI registry, we developed a risk score for predicting in-hospital mortality, comprising 8 readily available variables (respiratory rate of >35 breaths/min, cardiogenic shock, oxygen saturation of <93%, age of >55 ​years, infiltrates on chest x-ray, kidney disease, diabetes, and dyspnea). The risk of in-hospital mortality increased exponentially with increasing NACMI score category from 3.6% to 60%, in the lowest vs highest categories, respectively (Central Illustration).

Our model discriminated well in the derivation cohort (c-statistic ​= ​0.81) and compares well with similar models predicting outcomes in the setting of acute coronary syndrome.^9^^,^^10^ STEMI management in patients with COVID-19 requires an accurate model of identifying risk, as some may require intensive care unit beds, mechanical ventilation, and/or mechanical circulatory support—the very resources that are scarce during this pandemic. Emanuel et al^11^ suggest that fair allocation of the scarce medical resources during the pandemic requires scientifically derived consideration of prognosis with priority given to those with a higher likelihood of survival. The NACMI risk score gives health care professionals a tool to navigate difficult scenarios.

In-hospital mortality in the pre-COVID-19 STEMI registries ranges from 2% to 10%.^10^^,^^12^, ^13^, ^14^, ^15^ In a recent contemporary analysis from the United States, the in-hospital mortality of uncomplicated STEMI was 2% but increased substantially in patients with cardiogenic shock (23%), cardiac arrest (19%), or both (44%).^15^ Although viral illnesses are known to increase the incidence of myocardial infarction and death,^16^, ^17^, ^18^ the 28% in-hospital mortality observed in patients with COVID-19 and STEMI in the NACMI registry was higher than a contemporary cohort of patients without COVID-19 (mortality rate of 11%) from the same sites and a propensity-matched prepandemic cohort for a large regional STEMI registry (mortality rate of 4%).^1^ This disproportionally high mortality rate was similar to other COVID-19 STEMI registries. For example, Saad et al,^4^ using an administrative database of >700 US academic hospitals, demonstrated mortality rates of 15% in patients with COVID-19 and out-of-hospital STEMI and 77% in patients with COVID-19 and in-hospital STEMI. In the international COVID-19–acute coronary syndrome registry, 144 patients from 55 international centers who underwent invasive coronary angiography in the setting of STEMI and confirmed or suspected COVID-19 had an in-hospital mortality rate of 22.9%.^3^ Moreover, these rates are higher than those of hospitalized patients with COVID-19 but without STEMI, reported between 7% to 10%.^19^^,^^20^ Similar to the study by Saad et al,^4^ NACMI patients already hospitalized with COVID-19 who developed STEMI had a high mortality rate. However, the in-hospital presentation was not included in our final multivariable regression model as the other predictors had more significant *P* values (Supplemental Table S1).

A more granular look at the NACMI cohort may reveal why these patients have high mortality rates compared with the benchmark analyses of STEMI populations pre-COVID era, such as Thrombolysis in Myocardial Infarction (TIMI)^14^ and the more contemporary analyses by Omer et al.^15^ Patients enrolled in the NACMI registry have the following: (1) higher non-White representation (54% vs 6% in TIMI), (2) higher rates of diabetes (46% vs 14% and 15% in TIMI and the report by Omer et al,^15^ respectively), and (3) higher rates of cardiogenic shock (16% vs 3% and 9% in TIMI and report by Omer et al,^15^ respectively). Furthermore, in our initial description of the NACMI registry, significantly higher rates of patients with STEMI and COVID-19 were not referred for angiography compared with a contemporaneous cohort of patients with STEMI in whom SARS-CoV-2 infection was ultimately ruled out (22% vs 4%).^1^

### Implication

Similar to risk prediction models GRACE^21^ and TIMI,^14^ the NACMI model identifies variables such as age, cardiogenic shock, and renal dysfunction as important clinical determinants of in-hospital mortality. However, the identification of markers of respiratory involvement/distress (such as hypoxemia, dyspnea, and infiltrates on chest x-ray) is unique. Although respiratory failure is an important general predictor of risk in patients with COVID-19, the NACMI registry adds to the traditional STEMI risk estimate^21^^,^^22^ in providing additional perspective on the relationship between respiratory distress/failure and in-hospital mortality for STEMI in patients with COVID-19. Importantly, respiratory variables collectively accounted for 41% of the NACMI risk score. It has been suggested that this is a unique STEMI phenotype in patients with COVID-19, in which cardiac disease is a secondary manifestation of a systemic pulmonary disease pattern,^23^ thus making the management of STEMI challenging. Although dyspnea on presentation and hypoxemia are known to be poor prognosticators,^19^^,^^20^ accompanying evidence of increasing respiratory acuity (such as tachypnea > 35 breaths/min) were associated with an increase in mortality in our cohort. Kidney disease has been reported in patients with severe COVID-19 with a prevalence of elevated serum creatinine level in 14.4% of the patient population and is an independent risk factor for in-hospital death.^24^ Our analysis found a slightly higher rate of increased serum creatinine on presentation but a similar independent effect on mortality, indicating a systemic vascular injury in our patient population. These findings likely reflect the interplay of the multiple pathobiological pathways that are concomitantly activated in patients with STEMI and COVID-19, often culminating in multiorgan involvement and increased in-hospital mortality.

Similar to previous research,^25^, ^26^, ^27^ advanced age and diabetes emerged as strong predictors of in-hospital death in the NACMI registry. In addition to being directly linked with increased cardiovascular risk,^28^ other pathways in which diabetes leads to poor outcome include a higher incidence of “diabetic lung” associated with decreased lung volume and reduced pulmonary diffusing capacity^29^ and an exaggerated inflammatory response associated with an increased renin-angiotensin system activation.^30^

### Limitations

There are noteworthy limitations to our study. First, our sample size was too small to allow for validation in a subset of our cohort; efforts to externally validate our findings will be needed. Second, fibrinolysis was uncommon in our registry (4%); therefore, that mode of reperfusion was excluded in our analysis as a predictor of mortality. Although this may be appropriate for North American practice in which fibrinolysis is uncommon, it may not be generalizable to other parts of the world in which it is more common. Third, the NACMI mortality risk score does not differentiate between cardiovascular and noncardiovascular mortality. In fact, it is difficult to determine the specific cause of death, a limitation that has been recognized in critically ill patients.^31^ Fourth, the elevated respiratory rate must be considered with great context, as patients may be intubated and sedated, which could mask this finding. The lack of vaccination status also limits the generalizability of these results.

Finally, despite a significant mortality rate in our patients who never had PCI, we chose not to include the “no-PCI” group in our model because it occurred after the presentation of STEMI. The no-PCI group represents a heterogenous group of patients who never had angiograms and/or had angiograms without clarity on the status of culprit vessel disease. We have previously shown a high preponderance of no-culprit lesions^32^ in COVID-19, making PCI an imperfect marker in this patient population. A core laboratory angiographic substudy is underway to specifically address the angiographic findings in these patients with correlation to hospital outcome. In addition, PCI for a cohort of patients with STEMI and COVID-19 infections may have been perceived as futile due to patients’ multiple comorbidities, such as concomitant mechanical ventilation and/or profound sepsis. Therefore, there is a strong bias for healthier patients going for cardiac catheterization laboratory, leading to a faulty conclusion that PCI is responsible for better survival.

## Conclusion

The mortality risk in patients with COVID-19 and STEMI can be assessed with a risk score comprising readily available clinical variables and may assist clinicians in the allocation of scare resources during the pandemic.

## Figures and Tables

**Supplemental Figure S3:**