# Supplementary material for: North American COVID-19 Myocardial Infarction (NACMI) Risk Score for Prediction of In-Hospital Mortality
Source: J Soc Cardiovasc Angiogr Interv. 2022 Jul 9;1(5):100404. doi: 10.1016/j.jscai.2022.100404 (PMC9270689; doi:10.1016/j.jscai.2022.100404)
Supplement: Supplemental Table S1 [file mmc1.docx]

**Supplemental Table S1-** Association of Clinical and Management Characteristics with Death in patients with COVID-19 and STEMI

|  | **Odds Ratio** | **95% CI** | **p-value** | **Patients (%)** | **Mortality Rate (%)** |
| --- | --- | --- | --- | --- | --- |
| **Patient Demographics** | | | | | |
| Age>55 | 3.38 | 1.84 – 6.21 | <0.001 | 74 | 33 |
| Nursing Home residence | 2.41 | 0.91 – 6.40 | 0.078 | 4 | 47 |
| Female Sex | 1.12 | 0.70 – 1.79 | 0.636 | 28 | 29 |
| White Race | 0.74 | 0.48 – 1.14 | 0.170 | 45 | 24 |
| **Risk Factors and Co-Morbidities** | | | | | |
| Kidney disease* | 2.85 | 1.77 – 4.59 | <0.001 | 23 | 45 |
| Diabetes | 1.99 | 1.30 – 3.06 | 0.002 | 43 | 36 |
| Hypertension | 1.81 | 1.09 – 3.01 | 0.022 | 72 | 31 |
| Hx Stroke | 2.04 | 0.99 – 4.22 | 0.054 | 8 | 42 |
| Previous CABG | 1.96 | 0.77 – 4.99 | 0.160 | 5 | 42 |
| Smoking Hx | 0.68 | 0.44 – 1.06 | 0.088 | 43 | 24 |
| Current Smoker | 0.60 | 0.33 – 1.08 | 0.088 | 19 | 20 |
| Previous PCI | 0.67 | 0.33 – 1.35 | 0.258 | 12 | 21 |
| Hx CAD | 1.07 | 0.65 – 1.77 | 0.783 | 23 | 29 |
| Previous MI | 0.78 | 0.39 – 1.54 | 0.472 | 12 | 24 |
| Dyslipidemia | 1.08 | 0.70 – 1.66 | 0.729 | 42 | 29 |
| **Presentation** | | | | | |
| Resp Rate>35 | 6.75 | 2.53 – 18.03 | <0.001 | 5 | 70 |
| O2 sat<93 | 5.50 | 3.28 – 9.21 | <0.001 | 19 | 59 |
| Shock Pre-PCI | 4.95 | 2.81 – 8.73 | <0.001 | 14 | 59 |
| Infiltrates on Chest Xray | 2.84 | 1.84 – 4.40 | <0.001 | 43 | 40 |
| In-Hospital Presentation | 2.62 | 1.22 – 5.61 | 0.013 | 7 | 48 |
| Dyspnea | 2.09 | 1.36 – 3.23 | 0.001 | 49 | 35 |
| Out of hospital cardiac Arrest | 2.09 | 1.03 – 4.23 | 0.041 | 8 | 43 |
| Pleural effusion on Chest Xray | 1.59 | 0.79 – 3.18 | 0.193 | 9 | 37 |
| Cardiomegaly on Chest Xray | 1.40 | 0.67 – 2.91 | 0.370 | 8 | 34 |
| **Management**** | | | | | |
| Mechanical Ventilation | 7.92 | 4.88 – 12.84 | <0.001 | 27 | 60 |
| No PCI | 2.67 | 1.72 - 4.13 | <0.001 | 36 | 53 |

CABG, coronary artery bypass grafting; PCI: Percutaneous Coronary Intervention; STEMI, ST-segment elevation myocardial infarction.

*Kidney disease was defined as Creatinine > 1.5mg/dL on presentation

**Management variables not included in main manuscript risk score
