# Supplementary material for: North American COVID-19 Myocardial Infarction (NACMI) Risk Score for Prediction of In-Hospital Mortality
Source: J Soc Cardiovasc Angiogr Interv. 2022 Jul 9;1(5):100404. doi: 10.1016/j.jscai.2022.100404 (PMC9270689; doi:10.1016/j.jscai.2022.100404)
Supplement: Supplemental Table S2 [file mmc2.docx]

**Supplemental Table S2** - Multivariable Analysis of in-hospital Mortality in patients with COVID-19 and STEMI with Management Variables Included

| Multivariable Analysis |  |  |  |  |  |
| --- | --- | --- | --- | --- | --- |
|  | Integer Score | Coefficient | Odds Ratio | 95% CI | p-value |
| Mechanical Ventilation | 11 | 1.68 | 5.35 | 3.00 – 9.54 | <0.001 |
| Resp rate>35 | 10 | 1.63 | 5.11 | 1.44 – 18.11 | 0.012 |
| Shock pre-PCI | 6 | 1.07 | 2.92 | 1.47 – 5.80 | 0.002 |
| Age>55 | 6 | 1.06 | 2.88 | 1.41 - 5.91 | 0.004 |
| O2 Sat<93 | 5 | 0.96 | 2.60 | 1.41 – 4.81 | 0.002 |
| Diabetes | 5 | 0.82 | 2.28 | 1.33 – 3.92 | 0.003 |
| In-hospital presentation | 4 | 0.80 | 2.22 | 0.90 – 5.48 | 0.083 |
| Infiltrates | 4 | 0.57 | 1.78 | 1.04 – 3.04 | 0.036 |
| No PCI | 4 | 0.70 | 2.00 | 1.17 – 3.43 | 0.011 |

Resp, Respiratory; PCI, Percutaneous coronary intervention; Sat, Saturation
